# Who Shall Prepare Our Food?

**Published:** 1899-01

**Authors:** 


### Who Shall Prepare Our Food?

The right preparation of food is certainly one of the great problems of the day. That a very great deficiency in this particular exists, no one familiar with the common affairs of life would for a moment deny. In the preparation of by far the largest portion of our food, its nutrient properties are in a more or less degree impaired, and are often well nigh destroyed. This occurs because of the proper appreciation of the importance of the subject, together with a want of knowledge of proper methods by those who prepare our food. Upon the proper preparation and use of our food depends our health, happiness and prosperity. It is of vastly more importance than the cut and style of our clothing, than the convenience of our homes, and than the aesthetic character of our immediate surroundings. Our intellectual, moral, and spiritual conditions are very greatly modified by the condition of our bodies, if the latter suffers the former necessarily so.

The fact of this deficiency has long been recognized by a few. The method of remedy has, however, not been sufficiently revealed to command the special attention of any. From certain intimations that have appeared recently a movement is being inaugurated that tend in the right direction, and if they are rightly prosecuted will result in the accomplishment of great good.

In our own city (Cincinnati) an effort is being inaugurated for the establishment of a training school for girls and women, whose business it is and will be to prepare food, that they may be trained thoroughly for this important work. This proposition is now being considered by a number of prominent persons, chiefly women, in this city, and is being specially fostered by a number of persons of position and influence, which gives encouragement, that practical results will be realized in the near future. One of our city papers, says: "If the plans go through, the great problem of domestic service may be in a fair way to be solved. It is proposed to secure for this school all necessary accommodations, appliances, and accessories for the skillful training of cooks, waitresses, house-keepers, etc. Such a school or training would be of the utmost importance, not only to those who are to be employees in the culinary department of our homes, but is as important to those who preside over these homes and who secure employees for their service. No woman is fully qualified and prepared to take charge of and conduct a home for a family, who is ignorant of the qualities and best methods of preparation of that upon which a family is to live.

Hardly anything is more unpleasant or sad than to see one, having in charge or presiding over a household, who is in a large measure and too often totally ignorant of the culinary art, and who is too often at the mercy of ignorant and presuming servants; not able to judge of the qualities of those they employ nor of the work they do. Not all housekeepers are thus deficient, but by far too many are, and in this respect the interest of those who have, or are to have the care of a home, should not be overlooked and provided for.

There are then two classes whose interest should be studied in this matter, those who are to be employees, or the mistress of the home, and those who are to be employed to do this important work for the household.

